# Multi-Omics Reveals Aberrant Phenotypes of Respiratory Microbiome and Phospholipidomics Associated with Asthma-Related Inflammation

**DOI:** 10.3390/microorganisms13081761

**Published:** 2025-07-28

**Authors:** Huan Liu, Zemin Li, Xu Zhang, Jiang-Chao Zhao, Jianmin Chai, Chun Chang

**Affiliations:** 1 Department of Respiratory and Critical Care Medicine, Peking University Third Hospital, Beijing 100191, China; jxleo@stu.pku.edu.cn (H.L.); john_wick@stu.pku.edu.cn (Z.L.); 2 Research Center for Chronic Airway Diseases, Peking University Health Science Center, Beijing 100083, China; 3 Tianjin Key Laboratory of Metabolic Diseases, Tianjin Medical University, Tianjin 301700, China; xuzhang@tmu.edu.cn; 4 Department of Animal Science, Division of Agriculture, University of Arkansas, Fayetteville, AR 72701, USA; jzhao77@uark.edu; 5 Guangdong Provincial Key Laboratory of Animal Molecular Design and Precise Breeding, College of Life Science and Engineering, Foshan University, Foshan 528011, China

**Keywords:** asthma, microbiome, sphingolipid, glycerophospholipid, inflammation

## Abstract

Respiratory microbiota and lipids are closely associated with airway inflammation. This study aimed to analyze the correlations among the respiratory microbiome, the airway glycerophospholipid–sphingolipid profiles, and airway inflammation in patients with asthma. We conducted a cross-sectional study involving 61 patients with asthma and 17 healthy controls. Targeted phospholipidomics was performed on exhaled breath condensate (EBC) samples, and microbial composition was analyzed via the 16S rDNA sequencing of induced sputum. Asthma patients exhibited significant alterations in the EBC lipid profiles, with reduced levels of multiple ceramides (Cer) and glycerophospholipids, including phosphatidylethanolamine (PE) and phosphatidylcholine (PC), compared with healthy controls. These lipids were inversely correlated with the sputum interleukin-4 (IL-4) levels. Microbiome analysis revealed an increased abundance of *Leptotrichia* and *Parasutterella* in asthma patients, both positively associated with IL-4. Correlation analysis highlighted a potential interaction network involving PA, PE, ceramides, *Streptococcus*, *Corynebacterium*, *Parasutterella*, and *Leptotrichia*. Specific alterations in airway microbiota and phospholipid metabolism are associated with asthma-related inflammation, supporting the concept of a microbiota–phospholipid–immune axis and providing potential targets for future mechanistic and therapeutic studies.

## 1. Introduction

Asthma is one of the most prevalent respiratory disorders, characterized by changing respiratory symptoms and airflow limitation [1]. According to the Global Burden of Disease Study, asthma affects approximately 295 million people worldwide [2]. Approximately 50% of adults with asthma exhibit eosinophilic, severe type two airway inflammation [3]. Despite the array of treatment options available, a substantial proportion of patients with asthma struggle with inadequate symptom control.

The pathogenesis of asthma is intricately linked to dysregulated metabolic pathways, with lipids emerging as increasingly valuable biomarkers for understanding the pathogenesis and severity of asthma [4,5,6]. Within the family of lipid molecules, phospholipids, including sphingolipids and glycerophospholipids, hold particular significance. Sphingolipids play pivotal roles in membrane structure and function. Ceramide, the central molecule in sphingolipid metabolism, originates from the condensation of two common cellular metabolites, serine and palmitoyl coenzyme A [7]. Glycerophospholipids are the predominant lipid type present within cell membranes. Their fundamental structure includes phosphatidic acid and phosphate-bound substituent groups, which can be divided into many classes, such as choline, cardiolipin, and inositol. Combined metabolomic and transcriptomic analyses have implicated glycerophospholipid and sphingolipid metabolism in house-dust-mite-sensitized asthmatic mice [8]. However, the mechanisms through which glycerophospholipids and sphingolipids affect asthma pathogenesis and physiological processes remain unclear.

Respiratory microbiota has been closely linked to allergic airway inflammation, including conditions such as asthma [9]. Recent research has highlighted the roles of specific bacteria, notably *Streptococcus*, *Haemophilus*, and *Moraxella*, in influencing local immune responses and modifying the severity and symptoms of airway inflammation [10]. Several clinical studies have suggested associations between respiratory microbiota and various asthma phenotypes [11,12,13]. For instance, three hallmark genes associated with type two inflammation, namely SERPINB2, POSTN, and CLCA1, have been linked to airway microbial composition [14]. Additionally, eosinophilic inflammation, Th17 gene expression, and steroid responsiveness have shown correlations with alterations in airway microbiota [15]. While both lipid metabolism and the airway microbiome have been independently implicated in asthma, their potential interplay remains poorly understood.

The relationship between respiratory microbiota, sphingolipids–glycerophospholipids, and the host’s lipid metabolism is complex, and changes in microbiota may affect the disease course by activating immunometabolism programs [16]. In gut microbiota studies, comorbidity with gestational diabetes has been associated with specific gut microbiota composition and circulating lipid profiles. Among them, the relative abundance of *Faecalibacterium* and *Prevotella* has been linked to circulating lipids, particularly lysophosphatidylethanolamine and phosphatidylglycerol. This interaction between circulating lipids and gut microbiota profoundly influences host physiology [17], whereas alterations in airway microbiota may be also associated with different phospholipid metabolism pathways.

In this study, we conducted a comprehensive analysis of the airway microbiome and phospholipid metabolome profiles in patients with asthma using a multi-omics approach, including targeted metabolomics of EBC and 16S rDNA gene sequencing of induced sputum. Our integrated analysis, which incorporates clinical features, such as metabolomics, microbiome data, and inflammatory cytokines, sheds light on novel connections between phospholipid metabolism, immune responses, and microbial pathways in patients with asthma. These findings could serve as potential targets for future interventions in asthma research.

## 2. Materials and Methods

### 2.1. Participants

In this cross-sectional study, we enrolled patients with asthma from the Department of Respiratory and Critical Care Medicine at Peking University Third Hospital. All the participants met the Global Initiative for Asthma (GINA) criteria for persistent asthma, had no respiratory infections, and had not used oral corticosteroids or antibiotics in the preceding four weeks. Patients with comorbid chronic obstructive pulmonary disease; bronchiectasis; pneumonia; obstructive sleep apnea hypoventilation syndrome; cancer; various acute or chronic respiratory failure; and severe cardiovascular, hepatic, or renal disease were excluded. Additionally, we randomly included 17 healthy individuals who were matched for sex, age, and body mass index (BMI). Healthy controls had no history of asthma, allergic diseases, or other chronic respiratory symptoms (cough/wheeze/dyspnea) in the past 12 months. They also exhibited normal spirometry (FEV_1_/FVC ≥ 0.8 and FEV_1_ ≥ 90% of the predicted value) and had not recently had a respiratory infection or used antibiotics within the past four weeks. The primary endpoint of this study was to evaluate the differences and correlations in airway microbiota and exhaled breath condensate (EBC) phospholipid metabolites between the asthma patients and the healthy controls and to assess their correlations with inflammatory phenotypes. The secondary endpoint was to perform an integrated network analysis of microbiota–metabolite–immune interactions. This study received approval from the Ethics Committee of Peking University Third Hospital (ethical approval for M2014071 was granted on 12 April 2016 and for M2019431 on 13 November 2019), and all the participants provided informed consent. This trial was registered with ClinicalTrials.gov (Trial Registration Number: NCT06327516).

### 2.2. Acquisition and Processing of Sputum Induction

According to previously published protocols [18], induced sputum was obtained from the participants following 20–30 min of nebulized inhalation of 3% hypertonic saline. Sputum plugs were separated from saliva, and subsequently they were incubated with an equal volume of 0.4% dithiothreitol in a 37 °C water bath and shaken for 30 min. This was followed by centrifugation at 600× *g* for 5 min to isolate sputum supernatants and cells. The cells were stained using Wright–Giemsa stain, and 200 inflammatory cells were counted and sorted. Sputum supernatant was subjected to enzyme-linked immunosorbent assay for the determination of matrix metalloproteinase-9 (MMP-9), interferon (IFN)γ, IL-4, IL-5, IL-13, IL-17A, and IL-10 (R&D Systems Ltd., Minneapolis, MN, USA). A portion of the sputum was employed for sputum cytology, whereas the remainder was stored at −80 °C for DNA extraction.

### 2.3. Respiratory Microbiome Sequencing and Bioinformatics

Microbial DNA was extracted from sputum samples using the QIAamp^®^ DNA Microbiome Kit (QIAGEN, Hilden, Germany) based on the manufacturer’s instructions as described previously [18]. The V3-V4 region of bacterial DNA was amplified with universal primers. PCR was performed using the KAPA HiFi Hotstart PCR kit. Sequencing libraries were determined using a Qubit 2.0 Fluorometer (Thermo Fisher Scientific, Waltham, MA, USA), and then sequenced on the Illumina HiSeq platform (Illumina, San Diego, CA, USA) for paired-end reads of 250 bp at the Realbio Genomics Institute (Shanghai, China). To avoid contamination from the reagents and the environment, negative controls, one in the DNA extraction step and another in PCR amplification, were included. No detectable bands were detected in agarose gels from the negative controls. Furthermore, we collected the datasets published by Wang J et al. [18] and re-analyzed the microbiome data using QIIME2. In brief, the software package QIIME2 (2023.5 release) [19] was used to analyze the next-generation sequencing data. First, the paired-end fastq files were imported into QIIME2 and were demultiplexed. Then, the Deblur program was used to remove low-quality and chimeric sequences, yielding high-quality amplicon sequence variants (ASVs) [20]. These high-quality reads were then classified with reference to the Greengene2 database [19]. The ASVs were classified based on 100% identity. Sequences were rarified to the minimum sequencing depth at 8750 reads to reducing the effects of sequencing depth on alpha and beta diversities. Alpha and beta diversities were calculated using QIIME2, and Analysis of Similarity (ANOSIM) was conducted to test the differences in beta diversity measures based on the unweighted Jaccard distance. The raw sequence data reported in this paper have been deposited in the Genome Sequence Archive in the National Genomics Data Center (GSA-Human: HRA007372) and are publicly accessible at https://ngdc.cncb.ac.cn/gsa-human (accessed on 22 July 2025). 

### 2.4. Analysis of Serum Cytokines

Following centrifugation, the serum samples were stored in a refrigerator at −80 °C after centrifugation. The levels of IFN-γ, IL-4, IL-5, IL-13, IL-17A, and IL-10 (R&D Systems Ltd.) were determined in the serum using the enzyme-linked immunosorbent assay (R&D Systems Ltd.) following the provided instructions.

### 2.5. EBC Collection

EBC was collected using the TURBO-DECCS exhaled breath condensate collector (Medivac, Parma, Italy) in line with the manufacturer’s guidelines. EBC collection adhered to the recent ATS/ERS recommendations [21]. During EBC collection, the participants underwent 10 min of tidal breathing, while wearing a nose clip. The EBC samples were aliquoted, immediately frozen, and stored at −80 °C until analysis.

### 2.6. Lipid Extraction

To extract lipids, 100 µL of EBC was mixed well with 400 µL of 75% ice cold methanol, and then vortexed for 2 min. Subsequently, 1 mL of methyl tert-butyl ether was added to the mixture and vortexed for 1 h at room temperature. Next, 250 µL of H_2_O was added, and the solution was centrifuged at 12,000× *g* 4 °C for 10 min. To dissolve the dried sample, 200 µL of 50% methanol was added, and the resulting solution was transferred into sample bottles.

### 2.7. Measurement of Phospholipids Using Liquid Chromatography–Mass Spectrometry (LC-MS)

LC-MS was employed to determine the EBC phospholipid profile. Analysis was conducted using an ultra-high-performance liquid chromatograph (Waters, Milford, MA, USA) equipped with a column of ethylene-bridged hybrid particles (BEH C18) measuring 2.1 mm × 100 mm with a particle size of 1.7 µm. The flow rate was set at 0.25 mL/min, and analysis was conducted at 25 °C. The mobile phase consisted of two components: A liquid, composed of 60% ethanol and 5 mmol/L acetic acid, and B liquid, which consisted of a 9:1 volume ratio of isopropanol to ethanol. The elution gradient was as follows: 0–3 min, 15% B liquid; 3–15 min, a linear increase in the amount of B liquid from 15–99%; 15–17 min, maintaining the amount of B liquid at 99%; 17–19 min, a linear decrease in the amount of B liquid from 99–15%; and from 19 to 20 min, maintaining the amount of B liquid at 15%. The mass spectrometer used was an AB Sciex 5500QTRAP equipped with an electrospray ionization source (Turbo V ESI). The scan mode employed was multiple reaction monitoring, and the ion source parameters were as follows: CUR = 30 psi, GS1 = 30 psi, GS2 = 30 psi, CAD = MEDIUM, TEMP = 650 °C, and IS = 4500 V (for positive ions).

### 2.8. Statistics

Normality of continuous variables was assessed using the Shapiro–Wilk test. For variables with normal distribution, group comparisons were performed using Student’s t-test or one-way ANOVA as appropriate. For variables not normally distributed, non-parametric tests, such as the Mann–Whitney U test or the Kruskal–Wallis test, were applied. The categorical data were compared using the Chi-square test or Fisher’s exact test. Correlations between the microbiome, the metabolites, and the clinical parameters were evaluated using Spearman’s rank correlation. False discovery rate (FDR) correction was applied for multiple testing where applicable. All statistical analyses were performed using Prism 6.0 (GraphPad Software, Inc., San Diego, CA, USA).

For the microbial data, alpha diversity was assessed using the Wilcoxon test. Linear discriminant analysis (LDA) effect sizes (LEfSe) were utilized to identify bacterial biomarkers of asthma, with an LDA absolute value over 3 considered as indicating differential bacteria between the two groups. Network analysis of bacterial interactions was calculated in the R package “psych” (version 2.3.6) using bacteria with a relative abundance over 0.1%. Spearman correlations among ASVs with a correlation co-efficiency over 0.7 or less than −0.7 and adjusted *p* values smaller than 0.05 were used for visualization using Cytoscape (version 3.8.2) software.

Bioinformatic analysis for metabolites was conducted using OmicStudio tools at https://www.omicstudio.cn/tool (accessed on 8 June 2025) and MetaboAnalyst 5.0 at http://www.metaboanalyst.ca (accessed on 8 June 2025). Orthogonal projections to latent structures discriminant analysis (OPLS-DA) and the t test were performed between the two groups, with the FDR adjusted *p* value < 0.05 and the Variable Importance in the Projection (VIP) > 1 being considered as significantly different metabolites.

Additionally, the “pheatmap” package in R was used to display the correlation between bacteria, metabolites, and phenotypes. Spearman rank correlation was used to evaluate the associations between the microbial genera, the phospholipid metabolites, the cytokines, and the clinical parameters. To control for false positives resulting from multiple comparisons, the Benjamini–Hochberg method was applied to adjust the *p*-values and control the false discovery rate (FDR). Only correlations with an absolute correlation coefficient |r| > 0.5 and an FDR-adjusted *p*-value < 0.05 were considered statistically significant and biologically meaningful. These thresholds were also used to construct correlation-based networks and heatmaps. Spearman’s analysis was performed using the “psych” package in R, and the network was visualized using Cytoscape (version 3.10.1). Moreover, the main factors (i.e., clinical signs) influencing airway microbiota or metabolites were assessed by Mantel and partial Mantel tests using the “ggcor” package of R. Briefly, Mantel tests were applied to compare the Euclidean distance matrices of the dissimilarities in microbiota or metabolites. The correlations between the driving factors and phylogenetic distance were estimated using a partial Mantel test.

## 3. Results

### 3.1. Demographic and Clinical Characteristics of Study Participants

A total of 78 participants were included in this study. Table 1 shows the clinical characteristics of the participants. The patients with asthma were comparable to the healthy controls in terms of age, sex, and BMI. None of the participants suffered from gastroesophageal reflux disease.

### 3.2. Differential Respiratory Microbiome in Patients with Asthma

**Figure 1:**
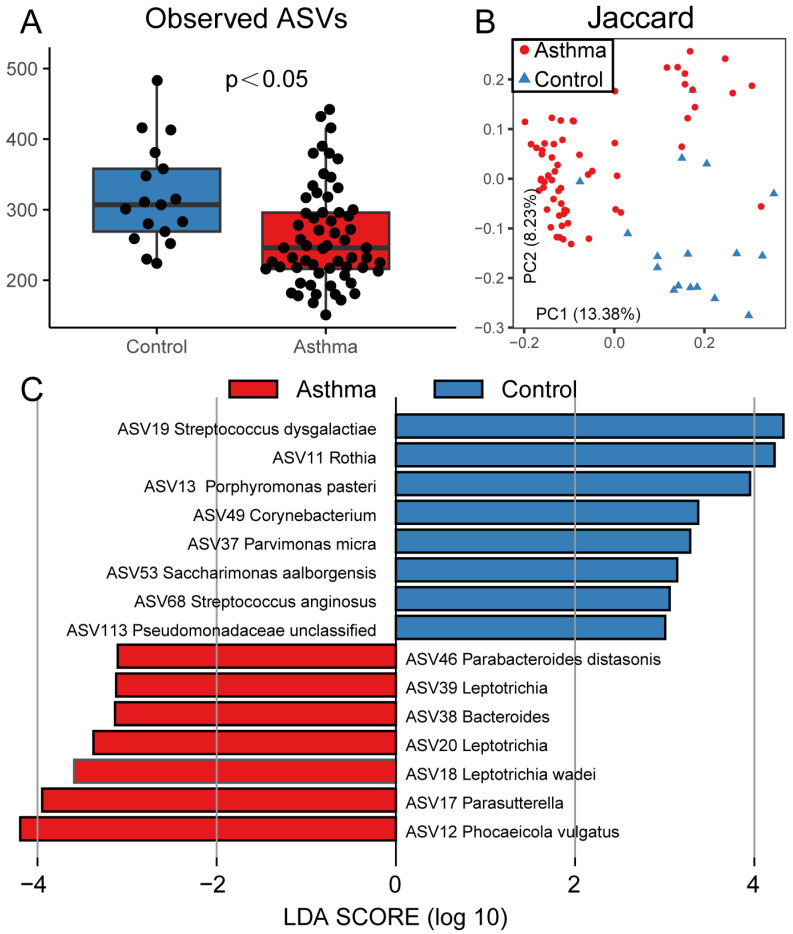
The microbial diversities of the healthy controls and the asthmatic patients. (**A**) The observed ASVs in the healthy controls were higher than in asthma patients. (**B**) Jaccard distance analysis. The control and asthma samples are differentiated by color (blue and red); (**C**) The bacterial biomarkers have been marked with LEfSe to differentiate between the healthy controls and the asthmatic patients.

To investigate variations in sputum microbiota among the asthmatic patients, we initially conducted 16S rDNA sequencing. Comparing the alpha diversity between the two groups, we observed that the number of observed ASVs was lower (*p* < 0.05) in the asthma patients (Figure 1A), while no difference in Shannon Index was observed (Figure S1). The results of beta diversity based on Jaccard distance (Figure 1B) indicated that the asthmatic patients’ samples were significantly different from those of the healthy controls in terms of airway microbiomes (ANOSIM: R = 0.5711, *p* < 0.001). Subsequently, we identified airway bacterial biomarkers in the patients with asthma using LEfSe at the ASV level (Figure 1C). The results revealed that the sputum of the healthy controls exhibited enrichment of several ASVs, including *Rothia* (ASV11), *Parvimonas* (ASV37), *Streptococcus* (ASV19 and ASV68), *Corynebacterium* (ASV49), *Porphyromonas* (ASV13), *Pseudomonas* (ASV113), and *Saccharimonas aalborgensis* (ASV53). In contrast, the sputum of the patients with asthma was enriched with ASVs, such as *Parabacteroides* (ASV46), *Leptotrichia* (ASV18, ASV20, and ASV39), *Bacteroides* (ASV38), *Phocaeicola* (ASV12), and *Parasutterella* (ASV17).

**Figure 2:**
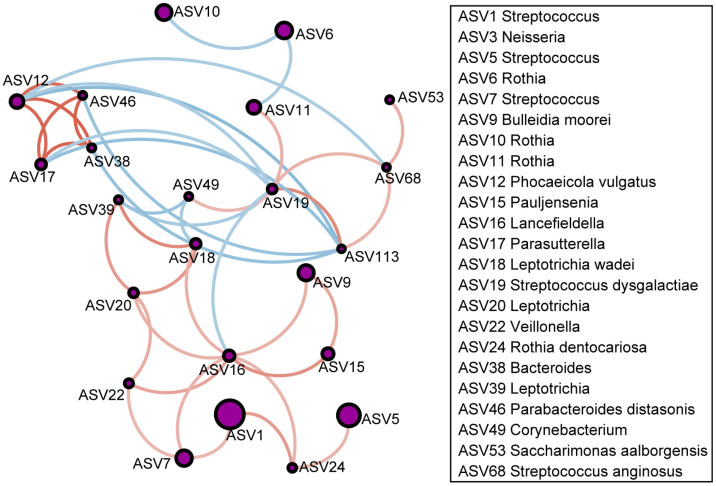
The co-occurrence networks in the microbial communities for the patients with asthma and the healthy controls. The orange edges indicate positive correlations between two nodes, while the blue edges indicate negative correlations.

Owing to the significant differences in the airway microbiomes between the asthma patients and the healthy controls, we further evaluated the microbial co-occurrence network for all the patients and the healthy controls (Figure 2). The results indicated several representative bacteria in the airway microbiome communities; *Streptococcus* (ASV19) in the co-occurrence network, which was enriched in the healthy controls, was negatively correlated with several ASVs enriched in the asthma group, such as *Leptotrichia* (ASV39) and *Parasutterella* (ASV17), and positively correlated with several taxa enriched in the healthy group, such as *Corynebacterium* (ASV49) and *Pseudomonas* (ASV113). Furthermore, *Leptotrichia* (ASV20) enriched in the asthma group in the co-occurrence network was found to be positively correlated with several microbiotas enriched in the asthma group, such as *Leptotrichia* (ASV18) and *Bacteroides* (ASV38).

### 3.3. Differential EBC Phospholipid Levels in Patients with Asthma

**Figure 3:**
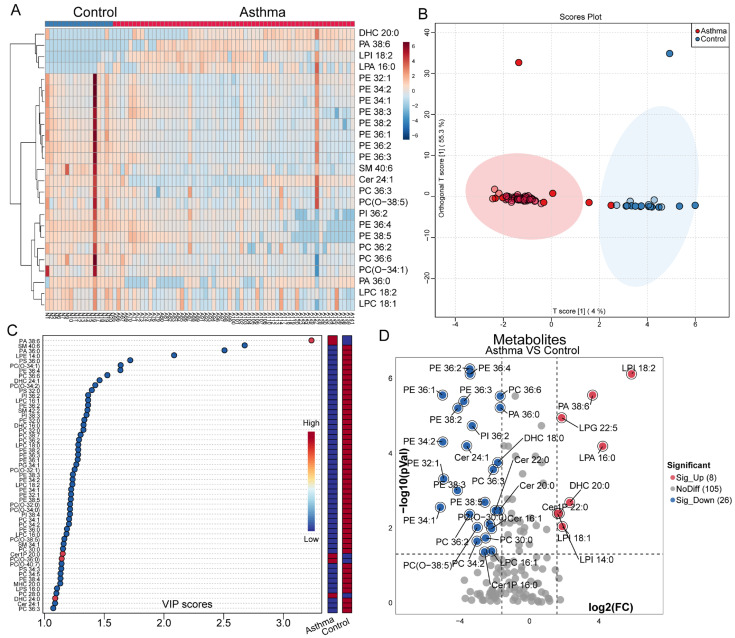
EBC metabolomics analysis: (**A**) Heatmap of EBC phospholipids comparing asthma patients and healthy controls; (**B**) OPLS-DA plot of EBC phospholipids for asthma patients and health control groups; (**C**) dot plots of phospholipid which VIP score≥1 based on OPLS-DA model in asthma and healthy controls; (**D**) volcano plot of identified metabolites based on FDR *p*-value ≤ 0.05 and |log_2_FC| ≥ 3.

A total of 249 different types of phospholipid were detected in the EBC of the asthmatic subjects and the controls, including 54 sphingolipids, such as Cer, Cer1P, DHC, MHC, SM, and Sulfatides, and 195 glycerophospholipids, such as PA, PC, PE, PG, PI, PS, LPA, LPC, LPE, LPG, LPI, and LPS. The top 25 phospholipid species ranked by the magnitude of the *p*-value are presented. Most of the phospholipid species showing significant differences, primarily glycerophospholipids, exhibited decreased abundance in the asthmatic patients. Only DHC20:0, SM40:6, and Cer24:1 displayed decreased abundance in the sphingolipid category among the asthma group (Figure 3A).

To further analyze the phospholipid profiles in the asthma and control groups, OPLS-DA scatter plots were generated. These plots demonstrated a clear separation trend in phospholipid levels between the patients with asthma and the healthy controls, indicating a significant alteration in the phospholipid profile of EBC in the patients with asthma (Figure 3B). Using a variable importance in projection (VIP) threshold of ≥1 in the OPLS-DA model, we identified 55 types of phospholipid metabolite (Figure 3C). We subsequently combined the FDR-corrected *p*-values and the fold change (FC) values from the volcano plot to identify potential biomarkers, revealing 34 differential phospholipid metabolites between the patients with asthma and the healthy controls. Among these, eight phospholipid species (including one sphingolipid and seven glycerophospholipids) were significantly upregulated, whereas twenty-six phospholipid species (including five sphingolipids and twenty-one glycerophospholipids) were significantly downregulated in the patients with asthma, as shown in Figure 3D, indicating that the significantly decreased sphingolipid species were all ceramides, such as Cer16:1, Cer20:0, Cer22:0, and Cer24:1, whereas the majority of the significantly less common glycerophospholipid species belonged to the PE and PC subclasses. Ultimately, 22 differential metabolites were identified between the asthma and healthy control groups based on the criteria of VIP ≥ 1, a false discovery rate (FDR), a *p*-value ≤ 0.05, and |log_2_FC| ≥ 3.

### 3.4. Correlation Between Differential Phospholipids in EBC and Differential Microbes in Sputum

**Figure 4:**
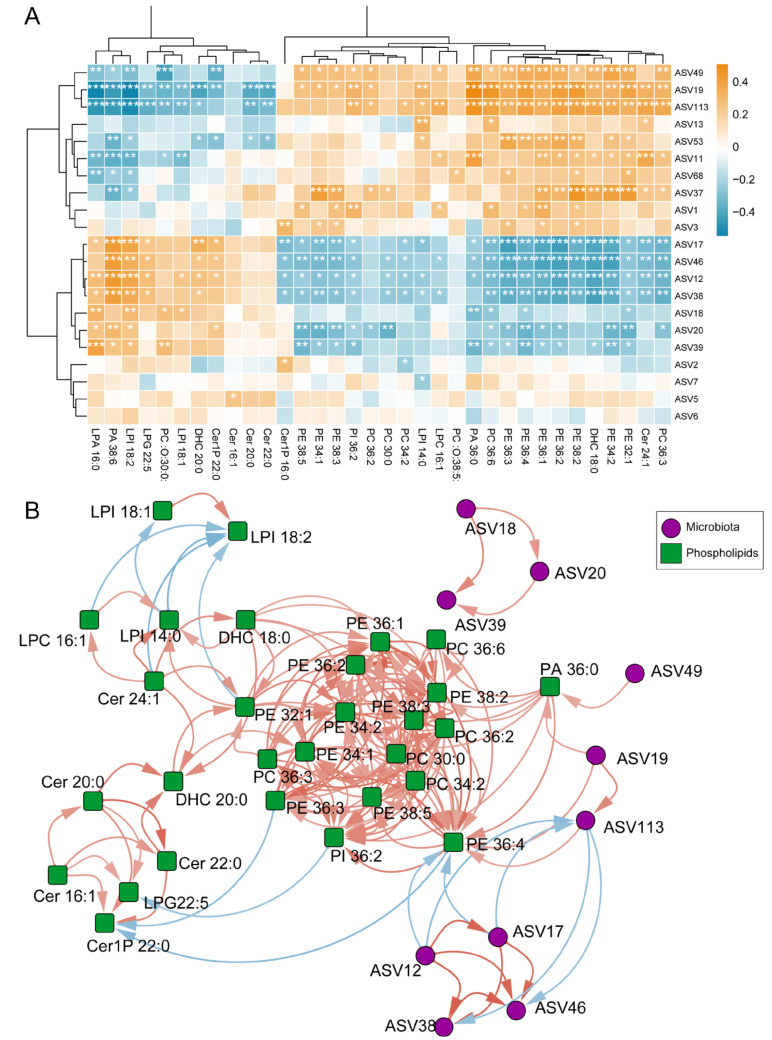
The correlation between differential phospholipids in EBC and differential microbes in the sputum: (**A**) A heatmap depicting the correlation between phospholipids in EBC and microbes in the sputum. (**B**) A correlation network between phospholipids in EBC and microbes in the sputum. The orange edges indicate positive correlations between two nodes, while the blue edges indicate negative correlations. (ASV39 *Leptotrichia*, ASV18 *Leptotrichia wadei*, ASV20 *Leptotrichia*, ASV49 *Corynebacterium*, ASV19 *Streptococcus dysgalactiae*, ASV113 *Pseudomonadaceae unclassified*, ASV17 *Parasutterella*, ASV12 *Phocaeicola vulgatus*, ASV38 *Bacteroides*, and ASV46 *Parabacteroides*). * *p* < 0.05, ** *p* < 0.01, *** *p* < 0.001.

After identifying the differences of both the sputum microbiota and the phospholipid profiles between the asthma patients and the control group, we then estimated the relationship between the respiratory microbiome and phospholipids to identify their roles in asthma. Further exploration of this correlation was conducted using Spearman’s correlation analysis (Figure 4A,B). Most of the ceramides and lysophospholipids negatively correlated with *Streptococcus* (ASV19), *Corynebacterium* (ASV49), and *Pseudomonadaceae* (ASV113) significantly increased in quantity in the healthy controls, and those that were positively correlated with *Parabacteroides* (ASV46), *Bacteroides* (ASV38), *Phocaeicola* (ASV12), and *Parasutterella* (ASV17) showed a significant increment in quantity in the patients with asthma. However, PE, PC, and several types of ceramide, such as Cer 24:1 and DHC 18:0, displayed an opposite correlation with the abovementioned bacteria, indicating that this phospholipids variation could be partially associated with differences in the respiratory microbiome.

Furthermore, the metabolites with a Spearman correlation coefficient >0.4 were selected for network construction alongside their associated microbiota (Figure 4B). PA36:0 was positively correlated with several glycerophospholipid-associated genera, including *Streptococcus* (ASV19) and *Corynebacterium* (ASV49), both enriched in the healthy controls. In contrast, PE36:4 was negatively correlated with asthma-enriched taxa, such as *Phocaeicola* (ASV12) and *Parasutterella* (ASV17). Both the PA36:0 and PE36:4 levels were significantly decreased in the asthma patients, and their correlations suggest a potential link between reduced glycerophospholipid levels and altered airway microbial composition in asthma.

### 3.5. Correlation of Microbes with Clinical Characteristics

To assess the relationship between airway microbiota and clinical features in asthma, we collected data on the ACT scores, the cytokine levels, and the eosinophil and neutrophil counts in both blood and sputum. Spearman correlation analysis was performed between these variables and microbial taxa with a mean relative abundance >0.001 (Figures S2 and S3).

Among the asthma patients, *Leptotrichia* (ASV39) showed a significant positive correlation with the ACT scores. While no significant associations were observed between peripheral blood eosinophils and microbial abundance, the sputum eosinophils were negatively correlated with *Phocaeicola* (ASV12), and the sputum neutrophils were positively correlated with *Parabacteroides* (ASV46) and *Bacteroides* (ASV38). These associations suggest that specific airway microbes may be linked to differential inflammatory cell recruitment.

**Figure 5:**
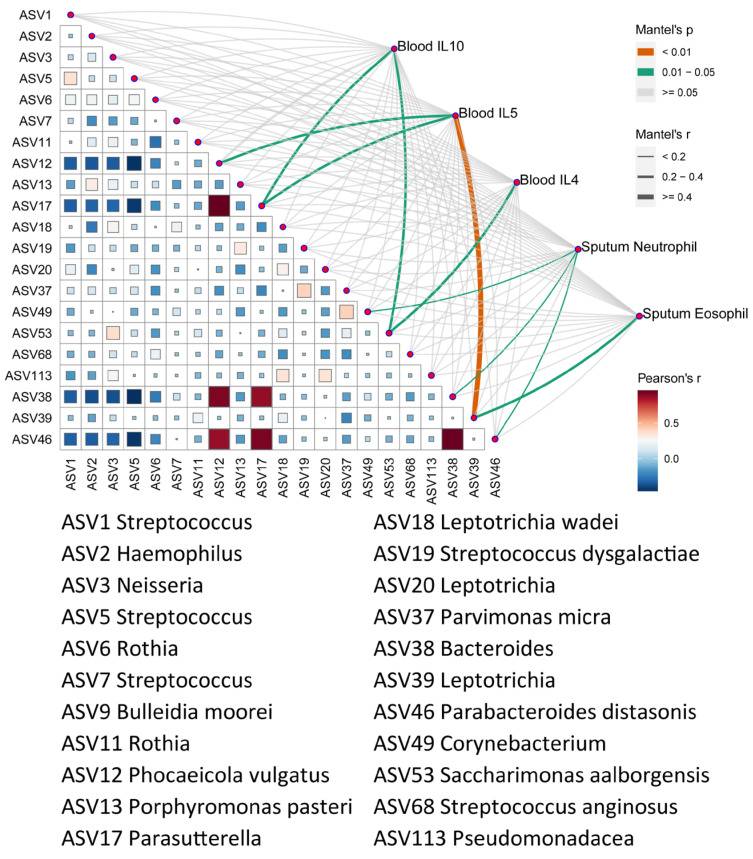
The effect of microbiomes on the clinical characteristics. Correlation of the respiratory microbial community and functional distance matrices with the clinical characteristics distance matrix, as determined using the Mantel’s test.

Additionally, we explored the correlations between the microbial profiles and inflammatory cytokines known to play key roles in asthma pathogenesis, including IFN-γ, IL-4, IL-5, IL-13, IL-17A, IL-10, and TGF-β1 [22,23,24]. Mantel test analysis revealed that the major microbial drivers were significantly associated with the cytokine profile in asthma (Figure 5). For example, *Leptotrichia* (ASV39), which was enriched in asthma, exhibited a strong correlation with the blood IL-5 levels. Similarly, *Phocaeicola* (ASV12) and *Parasutterella* (ASV17) were significantly correlated with IL-5 and IL-4. These findings suggest a potential association between asthma-related microbes and type two inflammatory responses. Further studies are warranted to elucidate the mechanisms underlying these microbe–immune interactions in asthma pathogenesis.

### 3.6. Correlation of Differential Phospholipids with Clinical Characteristics

**Figure 6:**
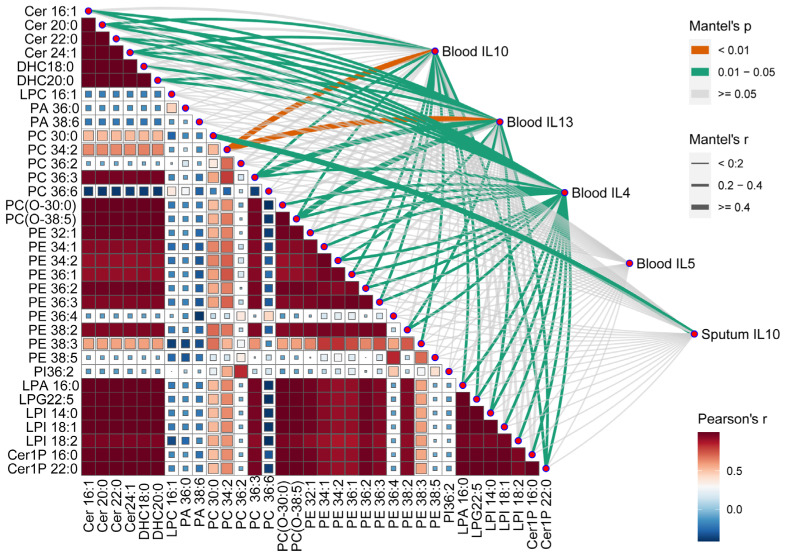
The effect of phospholipids on the clinical characteristics. Correlation of the phospholipids in EBC and functional distance matrices with the clinical characteristics distance matrix was determined using the Mantel’s test.

We then analyzed the correlation between the EBC differential phospholipids and the clinical characteristics (Figures S4 and S5). The sputum eosinophils were negatively correlated with DHC 18:0 and DHC 20:0, whereas the blood eosinophils were negatively correlated with DHC 20:0, suggesting that ceramides such as DHC 20:0 may contribute to the recruitment of airway eosinophils in patients with asthma. The key metabolic drivers jointly altering the inflammatory cytokines of asthma were evaluated by using the Mantel test (Figure 6). The most of ceramides, lysophospholipids, PE, and PC, could influence the cytokines in blood and sputum, which revealed a strong correlation with the phospholipids and the clinical characteristics. PE 36:1, PE 36:2, and PE 36:3 had a strong correlation with the cytokines related to IL-4 in the blood. Specifically, PC 34:2 had the greatest association with IL-13 and IL-10 in blood.

### 3.7. Correlation of Phospholipids, Microbes, and Clinical Characteristics

**Figure 7:**
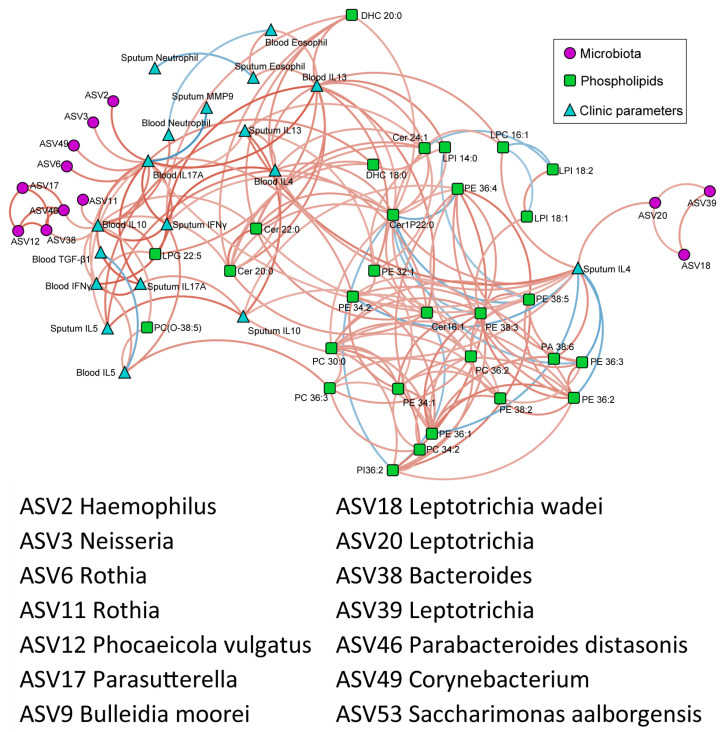
The correlation network among the phospholipids, the microbes, and the clinical characteristics (based on Spearman’s correlational analysis). The orange edges indicate positive correlations between two nodes, while the blue edges indicate negative correlations.

To investigate the interplay among the airway microbes, the phospholipid metabolites, and the clinical characteristics, we performed an integrated correlation network analysis (Figure 7). The network revealed three major modules comprising tightly connected microbial, metabolic, and clinical features. Among them, the phospholipids exhibited more extensive associations with the clinical parameters. For instance, PE 36:1, PE 36:2, and PE 36:3 were negatively correlated with sputum IL-4, while microbial taxa such as *Leptotrichia* (ASV20) were linked to multiple immunological markers, including sputum IL-4 and blood IL-10 and IL-17A, suggesting that both the PE species and *Leptotrichia* may represent key interface points between microbial metabolism and host immunity. In addition, Cer 20:0 and Cer 22:0 were positively associated with sputum IL-10, as well as taxa such as *Rothia* (ASV11), *Parabacteroides* (ASV46), and *Bacteroides* (ASV38). Collectively, these results indicate a coordinated pattern of interactions among phospholipids, microbes, and cytokine responses in the airway microenvironment of asthma patients.

## 4. Discussion

The emerging evidence suggests that asthma may be influenced by the microbiota–phospholipid axis, although the underlying mechanisms remain unclear. Our study expands the current knowledge by revealing several novel findings. First, we identified significant alterations in phospholipid metabolism in EBC from the asthma patients, including marked reductions in the levels of phosphatidylethanolamines (PEs), phosphatidylcholines (PCs), long-chain ceramides (LCCs), and very-long-chain ceramides (VLCCs), which have not been previously reported. Second, 16S rDNA sequencing of the sputum samples revealed distinct microbial profiles between the asthma patients and the healthy controls. Finally, by integrating the phospholipid, microbiome, and clinical data, we uncovered a network of associations that may contribute to understanding the microbiota–phospholipid–asthma axis.

Bacterial colonization of the airways begins at birth and stabilizes early in life [10,25]. The respiratory microbiota in patients with asthma has caused extensive concern [10,26]. However, certain bacterial infections may be protective against asthma [27]. In the current study, the levels of various bacteria were reduced in the sputum of the patients with asthma, including *Streptococcus*, *Corynebacterium*, *Porphyromonas*, *Pseudomonas*, *Rothia,* and *Parvimonas*. However, *Parabacteroides*, *Lachnospiraceae*, *Leptotrichia*, *Bacteroides*, and *Parasutterella* were found to be enriched in the patients with asthma, consistent with the previous findings [28,29,30]. Notably, *Leptotrichia* positively correlated with the ACT scores, suggesting a potential protective role in asthma control. The MMP-9 levels negatively correlated with *Corynebacterium*, which was downregulated in the asthma patients. Prior studies also link *Corynebacterium* to reduced nasal inflammation and a lower risk of acute exacerbations in severe pediatric asthma [31,32], indicating a probably protective role in patients with asthma. Similarly, the amount of *Porphyromonas* was reduced in the sputum of children with acute asthmatic exacerbation [33].

EBC derived directly from the airway offers valuable insights into respiratory diseases [34]. In this study, targeted phospholipidomics revealed significant alterations in multiple sphingolipids and glycerophospholipids in the EBC of asthma patients, with notable reductions in the amount of various PEs, PCs and ceramides. Beyond their structural roles in cell membranes, PE and PC are involved in diverse disease processes. Glycerophospholipid metabolism is also closely linked to the pathogenesis of childhood persistent wheezing (PW) [35]. Consistently, targeted lipidomics in a guinea pig asthma model showed decreased levels of 22 plasma saturated and unsaturated long-chain PCs [36]. We also observed that type two cytokines in the serum and the sputum negatively correlated with several PEs and PCs, suggesting these lipids may have protective roles in asthma. However, the underlying mechanisms must be clarified. Notably, modulating airway metabolites—such as through inhaled formulations—may offer a novel therapeutic approach.

We found the phospholipids and the microbiomes shared a strong correlation. Moreover, it has been reported that glycerophospholipids and sphingolipids contribute to the structural integrity of cell membranes and play crucial roles in cellular signal transduction and metabolism. The microbiome can produce large amounts of phospholipids via de novo biosynthesis and modification of host substrates. Microbially derived lipids have been shown to exert structural and signaling functions, influencing host cells through metabolic and immune pathways [17]. Microbial lipids are directly sensed by the host, modulating innate and adaptive immune pathways and regulating metabolic pathways. These regulations can affect the progression of chronic inflammation, autoimmune disease, cardiovascular disease, and metabolic syndrome. Likewise, respiratory microbiota may affect the host’s health via metabolism [37].

The association of glycerophospholipids with airway microbes has not yet been studied in chronic respiratory diseases. However, some studies have shown that hypoxia-induced *Desulfovibrio*-derived PCs and PEs in the gut promote γδ T-cell activation, contributing to intestinal injury [38]. In our study, asthma-associated bacteria such as *Leptotrichia* were negatively correlated with the glycerophospholipids, particularly PE36:1, PE36:2, and PE36:3. Notably, IL-4 was negatively correlated with PE and positively with *Leptotrichia*, suggesting a potential association among *Leptotrichia*, the PE levels, and type two inflammation. Previous studies have linked *Leptotrichia* with asthma [39], and PE38:1 has been proposed as a biomarker distinguishing asthma patients from healthy controls, possibly linked to serum IgE levels [40]. While the mechanistic role of PE in asthma remains unclear, it may be involved in redox balance and Th2 inflammation [41]. We also observed that *Parabacteroides* and *Bacteroides*, enriched in the asthma group, were negatively associated with PEs, Cer24:1, and DHC18:0, but positively associated with ceramides, such as Cer20:0, Cer22:0, and Cer1P22:0. Sphingolipids play roles in cell structure and signaling [42] and may originate from food intake, host synthesis, or microbial metabolism. *Bacteroides* species can produce sphingolipids that influence host lipid metabolism and immune signaling pathways in the gut [43]. Although their effects on invariant natural killer (iNKT) cells appear gut-specific and may not directly impact asthma [44], studies on germ-free mice colonized with sphingolipid-deficient bacteria have shown shifts in host ceramide levels, suggesting microbiota-derived sphingolipids may be linked to host metabolic and immune regulation [45]. Further studies are needed to clarify whether such associations extend to asthma.

In this study, one notable limitation is the relatively small number of healthy controls, particularly the low proportion of males. This imbalance resulted from the strict eligibility criteria for healthy participants, including the absence of chronic symptoms, respiratory infections, and recent medication use, which made recruitment challenging. Although only 3 of the 17 healthy controls were male compared to 21 of the 61 asthma patients, a Chi-square test showed no significant difference in sex distribution between the two groups, indicating acceptable group matching. Nevertheless, the limited sample size, especially among the male controls and the non-T2 asthma patients, may have reduced the statistical power of subgroup analyses and should be addressed in future research. Moreover, the cross-sectional design of this study limits our ability to infer causal relationships between respiratory microbiota, phospholipid metabolism, and asthma-related inflammation. Future studies employing longitudinal cohorts or interventional designs will be necessary to determine whether alterations in microbial or metabolic profiles contribute to the development or progression of asthma phenotypes.

## 5. Conclusions

This study presents a comprehensive comparison of respiratory microbiota, phospholipid metabolism, and inflammatory cytokines between asthma patients and healthy controls. The key microbial taxa and phospholipid species showed distinct patterns in asthma and were associated with inflammatory markers. Glycerophospholipid and sphingolipid metabolites may serve as potential links between airway microbiota and the inflammatory phenotype of asthma. The observed associations suggest a complex interplay between respiratory microbes and host lipid metabolism, warranting further investigation into the microbiota–phospholipid axis in asthma.

## Figures and Tables

**Table 1:** Characteristics of study subjects.

Characteristics	Asthma Patients	Healthy Controls	*p* Value
No. of subjects	61	17	
Age (years)	47.3 ± 17.8	50.5 ± 6.4	0.475
Sex (male/female), no.	21/40	3/14	0.242
BMI (kg/m^2^)	24.5 ± 4.0	23.1 ± 3.3	0.193
ACT score	18.5 ± 5.8	ND	
Smoking history (smoker/never), no.	11/50	2/15	0.722
Gastroesophageal reflux history	0	0	ND
ICS Dose			
Low	26	ND	
Medium	18	ND	
High	7	ND	
FEV1	89.8 ± 18.6	106.7 ± 16.8	0.001
FEV1/FVC	72.3 ± 11.4	84.1 ± 7.0	0.000
Allergy test positive, no.	48	ND	ND
T2-high asthma	55	ND	ND
Blood Eosinophil (×10^9^/L)	0.3 ± 0.2	ND	ND
Blood neutrophil (×10^9^/L)	2.9 ± 1.2	ND	ND
Sputum total cells count (×10^6^/g)	2.0 ± 1.6	1.3 ± 0.8	0.065
Sputum Macrophage (%)	16.1 ± 20.5	5.4 ± 6.0	0.034
Sputum Lymphocyte (%)	0.7 ± 3.2	0.5 ± 0.8	0.650
Sputum Neutrophil (%)	63.3 ± 30.0	87.9 ± 6.2	0.000
Sputum Eosinophil (%)	18.2 ± 24.7	0.3 ± 0.5	0.004
Sputum IL-4 (pg/mL)	2.4 ± 1.6	ND	ND
Sputum IL-5 (pg/mL)	7.5 ± 7.3	ND	ND
Sputum IL-13 (pg/mL)	7.1 ± 5.4	ND	ND
Sputum IL-10 (pg/mL)	2.2 ± 1.6	ND	ND
Sputum IL-17 (pg/mL)	1.0 ± 0.7	ND	ND
Sputum IL-10 (pg/mL)	2.2 ± 1.6	ND	ND
Sputum IFN-γ (pg/mL)	7.8 ± 10.3	ND	ND
Sputum MMP-9 (pg/mL)	51.5 ± 44.7	ND	ND
Blood IL-4 (pg/mL)	5.4 ± 4.2	ND	ND
Blood IL-5 (pg/mL)	8.1 ± 8.0	ND	ND
Blood IL-13 (pg/mL)	12.1 ± 8.9	ND	ND
Blood IL-17 (pg/mL)	32.1 ± 5.8	ND	ND
Blood IL-10 (pg/mL)	10.1 ± 5.1	ND	ND
Blood IFN-γ (pg/mL)	12.8 ± 5.1	ND	ND
Blood TGF-β (pg/mL)	846.2 ± 337.7	ND	ND

Note: Data are presented as the mean ± SD unless otherwise indicated. Abbreviations: BMI, body mass index; ACT, Asthma Control Test; ICS, Inhaled Corticosteroids.

## Data Availability

The raw sequence data reported in this paper have been deposited in the Genome Sequence Archive in the National Genomics Data Center (GSA-Human: HRA007372) and are publicly accessible at https://ngdc.cncb.ac.cn/gsa-human (accessed on 22 July 2025).

## Supplementary Materials

The following supporting information can be downloaded at: https://www.mdpi.com/article/10.3390/microorganisms13081761/s1. The supplementary file includes supplementary Figures S1–S5. Figure S1. Microbial diversity of Shannon Index in healthy controls and patients with asthma. Figure S2. Heatmap of Spearman’s correlational analysis between microbiome and clinical characteristics. Figure S3. Network analysis of interactions between microbiome and clinical characteristics. Figure S4. Heatmap of Spearman’s correlational analysis between phospholipids and clinical characteristics. Figure S5. Network analysis of interactions between microbiome and clinical characteristics.
