# Supplementary material for: Multi-Omics Reveals Aberrant Phenotypes of Respiratory Microbiome and Phospholipidomics Associated with Asthma-Related Inflammation
Source: Microorganisms. 2025 Jul 28;13(8):1761. doi: 10.3390/microorganisms13081761 (PMC12388397; doi:10.3390/microorganisms13081761)
Supplement: Supplementary file 1 [file microorganisms-13-01761-s001.zip › microorganisms-3722213-supplementary.pdf]

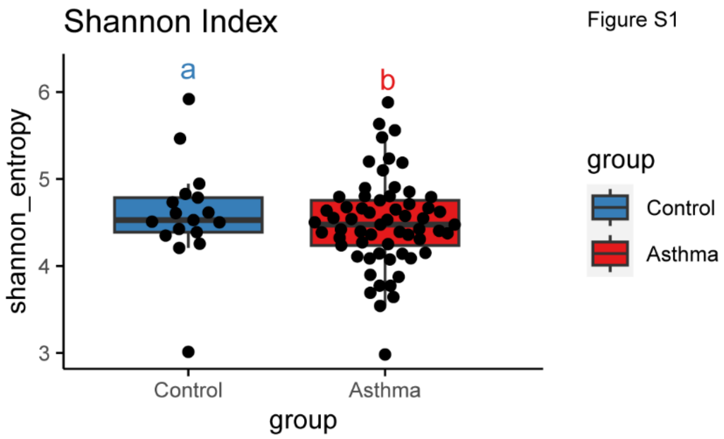

Figure S1. Microbial diversity of Shannon Index in healthy controls and patients with asthma.

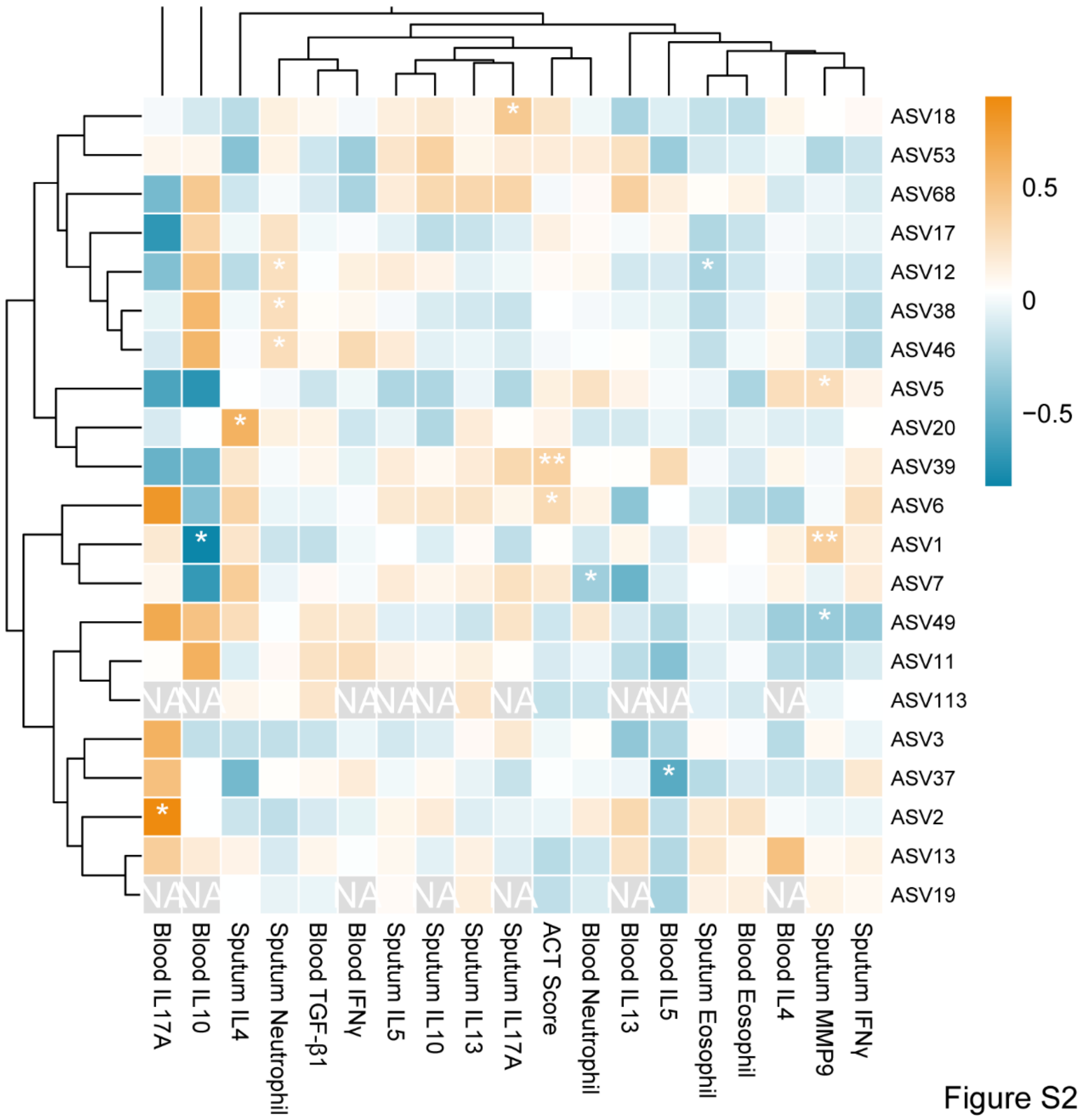

Figure S2. Heatmap of Spearman's correlational analysis between microbiome and clinical characteristics. Orange and blue colors indicate positive and negative correlations, respectively. *$p < 0.05$; **$p < 0.01$.

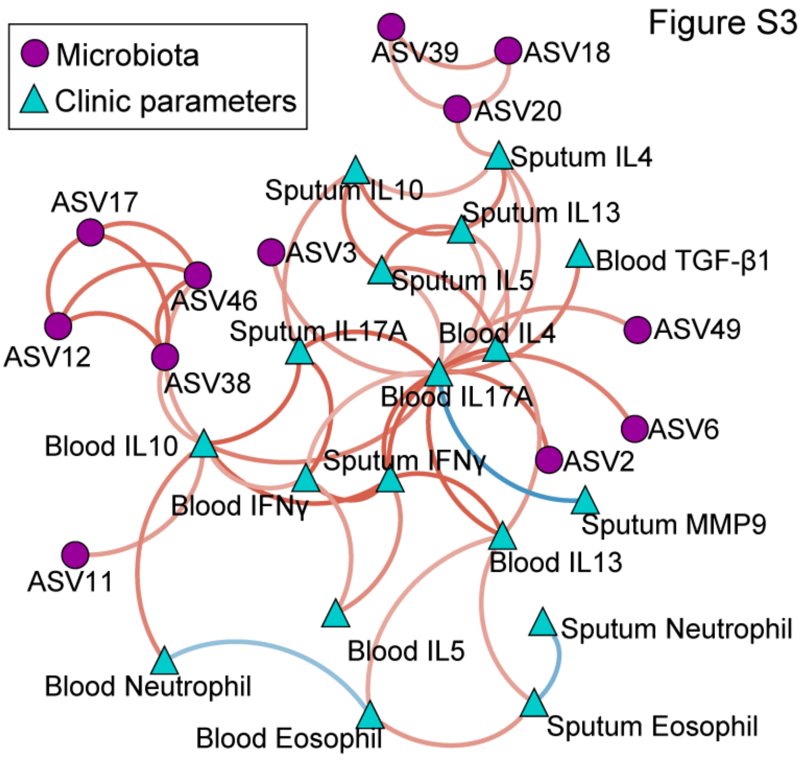

Figure S3. Network analysis of interactions between microbiome and clinical characteristics. Different colors denote 12 specific genera and 18 specific clinical characteristics within network. Each edge represents significant co-efficiency relationship (Spearman's rank correlation coefficient >0.4 or < −0.4).

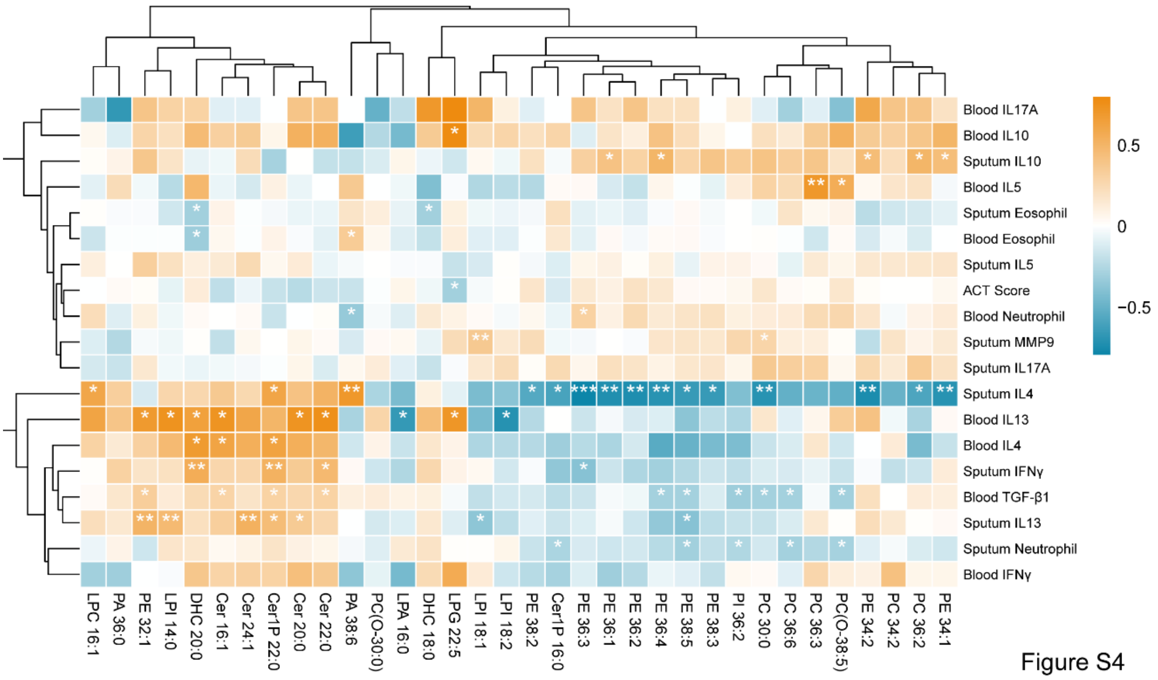

Figure S4

Figure S4. Heatmap of Spearman's correlational analysis between phospholipids and clinical characteristics. Orange and blue indicate positive and negative correlations, respectively. *$p < 0.05$; **$p < 0.01$; ***$p < 0.001$

Figure S5

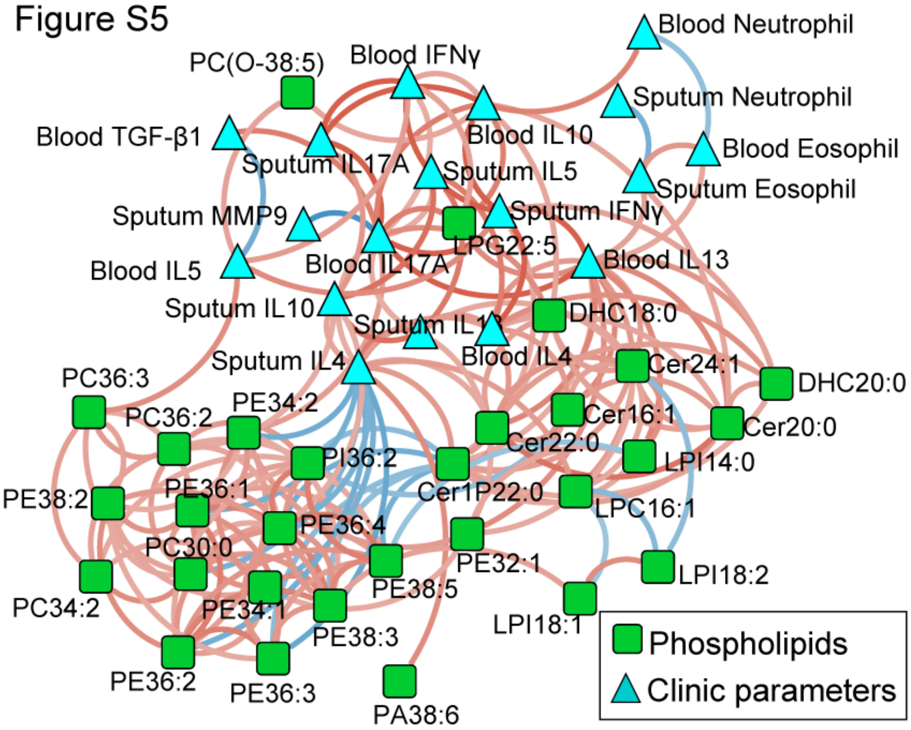

Figure S5. Network analysis of interactions between microbiome and clinical characteristics. Different colors denote 29 specific phospholipids and 18 specific clinical characteristics within network. Each edge represents significant co-efficiency relationship (Spearman's rank correlation coefficient >0.4 or < −0.4).
